# Air ambulance outcome measures using Institutes of Medicine and Donabedian quality frameworks: protocol for a systematic scoping review

**DOI:** 10.1186/s13643-020-01316-7

**Published:** 2020-04-02

**Authors:** Kristin H. Edwards, Gerard FitzGerald, Richard C. Franklin, Mark Terrell Edwards

**Affiliations:** 1 College of Public Health, Medical and Veterinary Sciences, James Cook University, 1 James Cook Drive, Townsville, Queensland Australia; 2 Queensland University of Technology, Victoria Park Rd, Kelvin Grove, Brisbane, Queensland Australia; 3 LifeFlight Retrieval Medicine Australia, Edward Street, Brisbane, Queensland Australia; 4 Queensland Health, Emergency Medicine Department Rockhampton, Rockhampton Base Hospital, Canning Street, Rockhampton, Queensland Australia

**Keywords:** Air ambulance, Outcome measures, Emergency system, Service delivery, Institutes of Medicine, Donabedian, Quality framework

## Abstract

**Background:**

Dedicated air ambulance services provide a vital link for critically ill and injured patients to higher levels of care. The recent developments of pre-hospital and retrieval medicine create an opportunity for air ambulance providers and policy-makers to utilize a dashboard of quality performance measures to assess service performance. The objective of this scoping systematic review will be to identify and evaluate the range of air ambulance outcome measures reported in the literature and help to construct a quality dashboard based on a healthcare quality framework.

**Methods:**

We will search PubMed, MEDLINE, CINAHL, Scopus, and Cochrane Database of Systematic Reviews (from January 2001 onwards). Complementary searches will be conducted in selected relevant journals. We will include systematic reviews and observational studies (cohort, cross-sectional, interrupted time series) in critically ill or injured patients published in English and focusing on air ambulance delivery and quality measures. Two reviewers will independently screen all citations, full-text articles, and abstract data. The study methodological quality (or bias) will be appraised using appropriate tools. Analysis of the characteristics associated with outcome measure will be mapped and described according to the proposed healthcare quality framework.

**Discussion:**

This review will contribute to the development of an air ambulance quality dashboard designed to combine multiple quality frameworks. Our findings will provide a basis for helping decision-making in health planning and policy.

**Systematic review registration:**

PROSPERO CRD42019144652

## Background

Air ambulances, both fixed and rotary wing, have become essential components in modern emergency healthcare systems. Drawing lessons from military conflict, the aim is to use fast transportation means to provide people with acute illnesses and injuries with access to centralized specialist care. The development of modern emegency medicine has provided a further opportunity to enhance the coordination and clinical standards available for the intended purpose of rapid response medical care provided to seriously sick and injured people [1].

However, the implementation of air ambulances are often tested in austere and unfamiliar situations [2–7], using limited and costly resources [8–11], and inequity of time [12, 13], distance [13–15], and accessibility [16–19]. Similar themes have emerged in multiple regions and countries (2:6, 8, 10:13). These developments of improved neurologic, cardiac, and trauma care pathways aim to reduce inequalities and ineffective efforts [8, 11–13, 18]. The increased utilization of air ambulance retrieval and transfers shows no sign of slowing [20, 21] which impacts on an already burdened emergency care system [22–25]. However, the drive to advance medical and aviation capability may tip the balance of future sustainability without a strategic health service plan which identifies and evaluates performance indicators [26]. Quality frameworks can provide structure to explore these indicators and form a basis for discussion. For these reasons, a scoping review was conducted to systematically map the range and nature of existing literature in this area, identify any existing gaps in knowledge [27], and synthesize the evidence in a framework.

The Institutes of Medicine (IOM) quality domains and Donabedian quality attributes are two generally accepted frameworks for health service performance measurements. IOM recognized six areas of improvement which are needed in response to inconsistent care across a rapidly changing health system [28]. The six areas were designed to encompass core needs of quality care:

- Effective: providing evidence-based care to all who could benefit and refraining from providing services to those not likely to benefit;
- Efficient: avoiding waste, including equipment, supplies, ideas, or energy;
- Safe: avoiding injury,
- Patient-centered: respectful and responsive to patient preferences, needs, and values;
- Timely: reducing waiting and delays for those that give and receive care; and
- Equitable: care which does not vary according to gender, ethnicity, geographic location, or socioeconomic status [28].

The IOM included recommendations for a system redesign to include the development of measures for assessing quality of care [28].

On the other hand, Dr. Avedis Donabedian believed that quality assessment should include three critical elements in healthcare delivery: structure, process, and outcome [29].

- Structure measure: material resources; facilities, equipment, human resources (number of personnel and their qualifications), and organizational structures (funding and reimbursement)
- Process measure: how healthcare is given and received as guided by policy, standards, and procedures
- Outcome measure: the effect of care and its impact on the health status of patients and populations

The structure of quality healthcare delivery needs to be built upon the material resources such as facilities, equipment, human resources (e.g., number of personnel and their qualifications), and organizational structures (e.g., funding and reimbursement). In turn, good structure increases the likelihood of good process, which includes how healthcare is provided and received through policy, standards, and procedures. The consequence of good structure and good process increases the likelihood of good outcome, effect of care, and health status of patients and populations [30].

Knowledge of the linkage between the three elements needs to be known before quality assessment can be conducted [30]. Donabedian includes caution around the certainty of assessing quality as it is often bound by the current strengths and limitations of clinical science, and outcomes are influenced by multiple factors, including the antecedent process of care [30].

As our combination of IOM and Donabedian quality framework has developed, other perspectives have emerged which further interpret these foundational criteria. These include further description of performance metric functions. Firstly, the US Government Performance Results Act 1993 (Section 2801) [31] outlines strategic performance metrics in four main categories: outcome, output, impact, and input.

- Outcome measure: “An assessment of the results of a program compared to its intended purpose”
- Output measure: “A tabulation, calculation, or recording of an activity or effort that can be expressed in a quantitative or qualitative manner”
- Impact measure: “A measure of the direct or indirect effect or consequence resulting from achieving program goals”
- Input measure: “A measure of the resources used to achieve an outcome (e.g., employees and funding)”

Secondly, the Agency for Healthcare Research and Quality (AHRQ) states clear differences between a process measure and a patient outcome measure [32, 33].

- Process measure: a healthcare-related activity performed for, on behalf of, or by a patient (e.g., readmission rates or discharge status) [33].
- Patient outcome measure: “a health state of a patient resulting from healthcare” (e.g., physiologic measures, radiology and lab results, and morbidity) [32]

For example, hospital staff shortage may delay a patient discharge, or inadequate patient teaching may lead to a readmission which may not pertain to patient physiology, but potentially, the process of the hospital or health system [34].

Finally, there is a pragmatic consideration in the identification and development of performance criteria. They must be measureable, meaningful, and manageable [35].

- Indicators should be able to be measured either through qualitative or quantitative means.
- Indicators should be meaningful in that they reflect quality of care and are considered important by both the clients and providers of health services and that they reflect the quality of services provided.
- Finally, indicators need to be manageable. Service providers need to be able to influence them and improve them. They also need to be efficient: data collection as a byproduct of the services provided and is not costly to collect.

These conceptual understandings help to create a combined framework which identify and evaluate the range and nature of air ambulance outcome measures of quality care.

### Objectives

The aim of this review will be to identify and evaluate the range and nature of air ambulance outcome measures reported in the literature and to construct a quality dashboard based on a sound conceptual framework.

The review will aim to address specific research questions:

1. What range of outcome measures are used in air ambulance literature?
2. What measurement instruments or tools were used to identify air ambulance quality of care?
3. Which air ambulance performance outcomes are utilized in our refined quality framework?
4. Can our quality framework create a performance dashboard for strategic improvement?

The PICO question is (P) patients critically ill or injured, (I) which require flight in a dedicated air ambulance, (C) we will not use comparison, and (O) air ambulance service outcome measures, constructed in a combined Institutes of Medicine and Donabedian quality dashboard.

## Methods

The review protocol has been registered within the International Prospective Register of Systematic Reviews (PROSPERO) ID no. CRD42019144652 and is being reported in accordance with the reporting guidance Preferred Reporting Items for Systematic Review and Meta-Analyses Protocol (PRIMSA-P) statement [36] (see checklist in Additional file 1).

Studies will be selected according to the following criteria: study design, setting, population, intervention, and outcomes

### Eligibility criteria

#### Study design

The review will include observational cohort, cross-sectional, longitudinal, interrupted time series, and systematic review studies. Randomized controlled, clinical controlled, and controlled before-after trials have numerous ethical constraints for air ambulance life and limb-saving interventions and cannot be balanced with a control group [37]. Therefore, these study designs will not be included in the search strategy.

#### Setting

The setting includes geographic (e.g., rural, urban, regional), multi-cultural, all levels of socioeconomic, and national/country of origin contexts.

The selected population (P) will include studies involving children, adolescents, and adults who are critically ill or injured (regardless of age or sex). The service intervention (I) we are considering are the following: patients which require flight on dedicated air ambulance missions/tasks (primary/scene/delayed primary/interfacility/interhospital/back-transfer), all aircraft type (helicopter/rotor-wing or fixed-wing), and crew mix (paramedic, nurse, doctor). The outcome (O) is first, to identify the range of air ambulance service outcome measures and their metric instruments represented in the literature, and second, to create a quality dashboard using a combined IOM and Donabedian framework, relevant to patients, providers, and policy-makers for future service improvement and planning.

Studies will be limited to articles published in English (from January 2001 onwards). These dates were chosen to coincide with the International Civil Aviation Organization (ICAO) recognition of new approaches to civil aviation safety risk and quality concerns in 2001 [38]. Exclusion criteria will include military studies, individual case studies, small case control studies outside of the general representative population (e.g., skier or snowboarder survival, SCUBA-related illness), equipment or device trials (e.g., active cooling apparatus for neonates, supraglottic airway devices), and drug or laboratory trials (e.g., diagnostic accuracy of serum lactate or mannitol dosing), as these are not relevant to the review.

### Data sources and search strategy

The PubMed search strategy will use relevant Medical Subject Headings (MeSH) terms (Additional file 2). For example, (1) Air ambulance “Fixed-wing aircraft or helicopters equipped for air transport of patients.” Subheadings may include classification, economics, ethics, history, legislation and jurisprudence, organization and administration, standards, statistics and numerical data, supply and distribution, and trends. No filters will restrict the MeSH major topics-only PubMed search builder options. (2) Outcome and Process Assessment (Health Care): “Evaluation procedures that focus on both the outcome and status (outcomes assessment) of the patient at the end of an episode of care—presence of symptoms, level of activity, and mortality; and the process (assessment, process)—what is done for the patient diagnostically and therapeutically.” Subheadings may include classification, economics, epidemiology, ethics, history, legislation and jurisprudence, methods, mortality, organization and administration, psychology, standards, statistics and numerical data, and trends. No filters will restrict the MeSH major topics-only PubMed search builder options. Search will include topics found below the MeSH hierarchy tree, if available.

The initial search strategy will include four databases commonly used in medical searches: PubMed, MEDLINE Ovid, CINAHL, Scopus, and Cochrane Database of Systematic Reviews from January 2001 onwards. A complementary search will include three relevant journals: *Air Medical Journal*, *Emergency Medicine Australasia*, and *Annals of Emergency Medicine*, as each has a dedicated section in pre-hospital retrieval and emergency medicine transport from January 2001 onwards. If necessary, we will contact authors to identify additional sources. A draft search for PubMed is included in Additional file 2.

### Study selection and data extraction

The selection process will use a pre-designed screening tool listing inclusion and exclusion criteria, and two authors (KHE, MTE) will independently examine study titles and abstracts following the PRISMA process. Screening will be managed in an Excel spreadsheet in descending chronological order of publication year and include complete citation. The authors will screen all citation titles and abstracts according to the selection criteria following the PRISMA process. The authors will record results with a colored Excel cell code and label extraction process. The cell color green means “yes,” red color cell “no,” or yellow color cell “maybe.” The authors will obtain full-text articles for potential relevance and then examine for eligibility. The authors will then assess and discuss the result for agreement. A third author will be included in the event of unresolved discrepancies. The authors will attempt to contact study authors in this event, to resolve uncertainties. Two authors will independently extract study data using a piloted form (Additional file 3) and checked for accuracy by a third author. Data extracted will include sample size, country(ies) study was performed, study setting, patient age range, pathology type or characteristic, air craft type, mission type, mission time interval, data source and type, crew type, intervention metrics, exclusion and inclusion criteria, limitations, comparison measures, primary and secondary (if available) outcome measures, funding source, and study results. Data extractors will not be blinded to study citations. There are no pre-planned assumptions or simplifications. Data extraction process steps will be maintained and managed using Microsoft Excel 2016. All publications will be managed using EndNote X8.

### Review of selected articles

Complete review of selected articles will be read and organized using a table format (Additional file 3).

### Outcomes and variations

The air ambulance outcome measures will further be defined according to the US Government Performance Results Act 1993 (Section 2801) [31]: “An assessment of the results of a program compared to its intended purpose.” Outcome measures could incorporate any assessment of this target (e.g., mortality and morbidity rates, adverse events, time-to-patient intervals, referral patterns or crew qualifications, dispatch criteria, or base proximity to tertiary facilities). The authors will attempt to interpret regional or national variations in terminology, if necessary (e.g., interhospital or delayed primary mission), and report the variations in glossary format, in the “Results” section of the review.

### Appraisal of evidence—risk of bias

Risk of bias quality will be assessed using ROBIS (risk of bias in systematic reviews) [39] and the Newcastle-Ottawa Scale (NOS) [40]. The ROBIS tool was chosen for the rigor in assessing the metabias in the systematic review process and the signaling questions as they relate to healthcare effectiveness (interventions) [40]. The NOS instrument was chosen for rigor in assessing the quality of nonrandomized studies. Three authors will independently assess the articles, one piloting and two with previous risk of bias appraisal experience. Disagreement between reviewers will be discussed until consensus is reached. Findings of the review will be included in the “Results” section and impact of bias, if any, in the “Discussion” and “Conclusion” sections. The ROBIS phase 2 applies signaling questions in four domains of key review processes at the study level: study eligibility criteria, identification and selection of studies, data collection and study appraisal, and synthesis and findings. ROBIS signaling questions are designed to “help assess specific concerns about potential biases” [39]. Each study level item will be assessed sequentially, not as “stand-alone units” [39]. ROBIS phase 3 process is at the outcome level, as a whole. This phase includes signaling questions and information to support the overall judgment of risk of bias. ROBIS assessment tools, for example ratings, signaling question explanations, and concerns for rating, will be used for guidance [39]. Answers to signaling questions are “yes,” “probably yes” (low concerns), “probably no,” “no” (higher concern), and “no information” (unclear). The table legend will include visual color and symbols for translation (Additional file 4). The NOS instrument assesses quality of selection (case definition, representativeness, case selection, control selection, control definition), comparability (case design or analysis), and outcome (assessment of outcome, length and adequacy of follow-up). Studies could be awarded a maximum score of 9 points. Studies with scores of 5 points or more are considered to be of moderate to good study quality [40]. NOS assessments will be presented in table format. Attempts will be made to contact authors for more information, if necessary. Appraisals will be made by three review authors based on ROBIS and NOS assessment guidelines. Disagreements will be resolved by discussion. If necessary, a fourth author will be consulted until consensus is reached.

### Planned approach to synthesis and analysis

Authors will summarize search results in a PRISMA study flow diagram [41] and by narrative synthesis in text and table format. Description of the five-phase narrative synthesis process will improve protocol transparency and reproducibility [42]. The authors will first summarize selected study variables in table format. Second, the authors will explore the findings and relationships in the combined IOM and Donabedian framework (e.g., how “time-to-patient” relate within the quality domains) (Table 1), using cognitive reconstruction [44] by collecting the outcome measures, then working backward to connect effect links in the framework. Third, the authors will discuss effect differences within the frameworks in a narrative format. Fourth, the authors will undertake thematic content analysis of selected article findings and relationships within the framework, using cognitive reconstruction [44], in a table format. Finally, the authors will present a visual dashboard diagram for patients, providers, and policy-makers to consider for future service improvement and planning.

**Table 1:** Proposed dashboard distribution strategy of air ambulance outcome measure examples in a combined IOM and Donabedian domains

IOM domain of quality	Donabedian measure type	Donabedian measure type	Donabedian measure type
	Structural examples	Process examples	Outcome examples
Effectiveness examples:	Appropriate HR(qualifications, quantity), facilities (proximity population, tertiary), equipment (ECMO), or funding structures which incorporate EBM	Appropriate guidelines or policy driven by EBM	Improved patient survival
Efficiency examples	Appropriate HR, facilities, equipment, or funding which minimize waste of equipment, ideas, or energy	Guidelines and policy which appropriate tasking to avoid over/under triage	Decrease in patient mortality and morbidity
Safety examples:	HR, equipment, facilities, funding structures which meet aviation and clinical safety regulation	Aviation and health procedures and guidelines which facilitate swift and safe departures	Patient survival; avoiding adverse events
Patient-centeredness examples	HR quantity and qualifications to meet patient and population-specific needs	Current standards of care to meet patient-specific needs	Survival; respecting patient values and preferences
Timeliness examples	Equipment, facilities, funding structure, HR quantity, and qualifications for timely assessment and treatment implementation	Active governance which monitor total system response time	Improved patient survival due to timely care
Equity examples	Equipment, facilities, HR, and funding structure to meet time/distance/patient variation	Appropriate policy, standards and/or procedures to meet needs of remote and disadvantaged communities	Patient survival across gender, ethnicity, geographic location, or socioeconomic status variations

*HR* human resources, *EBM* evidence-based medicine [43]

### Subgroup and sensitivity analysis

A subgroup or sensitivity analysis will not be undertaken in this review. The aim of the review is to identify and evaluate the range of air ambulance outcome measures reported in the literature, not to test the change effect of parameters.

### Publication bias

Publication bias will not be explored in this review, as the aim of the review is to identify the air ambulance outcome measures and tools, not the positive or negative results of outcomes.

## Discussion

Performance quality is able to be measured on many levels within the air ambulance health service: the frontline health providers, individual patient outcomes, the support systems (e.g., dispatch and triage), organizational structures (e.g., asset capability and availability), governance, and legislation. We acknowledge that quality is a challenging construct to define and measure in highly heterogenous, complex and interconnected emergency medical systems [45–48]. However, the first step is to explore air ambulance outcome measures as not an end, but rather the means to improving quality healthcare delivery [49]. The intent of this review is not to impose quality metric implementation, but rather introduce a generally accepted set of indicators which help to guage system-wide benchmarking and trend analysis [50]. Identification of the range of quality measurements reduces duplication, inconsistencies, and performance “gaps.” Evaluation of quality measure eliminates metric “cherry picking,” which highlights stakeholder’s self-interests [51]. Failure to identify meaningful outcome measures hinders the ability to recognize disparity and variations of care [50].

### Limitations

We acknowledge potential limitations of the review. These may include study inconsistencies in data collection and recording methods of critical information in the pre-hospital setting, such as field vital signs or response time [52]. Studies that use trauma registry data sources may have significant variability of definitions, standard measures, and case inclusion [52], which may influence study outcome. The authors acknowledge their limitations in language fluency, which are limited to English. Finally, there is a possibility to inadvertently miss relevant studies outside of our search strategy. Protocol amendments will be documented and available for open review on the PROSPERO website: https://www.crd.york.ac.uk/prospero/display_record.php?ID=CRD42019144652.

## Conclusion

In summary, dedicated air ambulance services provide a vital link for critically ill and injured patients to higher levels of care. The recent developments in modern emergency medicine create an opportunity for air ambulance providers and policy-makers to utilize a dashboard of quality performance measures. Our systematic review contains the first step toward the development of an air ambulance quality dashboard, designed to combine frameworks of the Institutes of Medicine and Dr. Avedis Donabedian and further refined using the Agency for Healthcare Research and Quality and the US Government Performance Results Act 1993, which aims to provide a basis for strategic health service planning.

## Supplementary information

**Additional file 1.** PRISMA-P 2015 Checklist.

**Additional file 2.** Medical Subject Headings (MeSH) terms.

**Additional file 3.** Review of selected article format.

**Additional file 4.** Risk of bias in systematic review using ROBIS sample.

## Data Availability

The datasets used and/or analyzed during the current study are available from the corresponding author on reasonable request.

## Abbreviations

AHRQ: The Agency for Healthcare Research and Quality
Donabedian: Dr. Avedis Donabedian
IOM: Institutes of Medicine
PROSPERO: International Prospective Register of Systematic Reviews
ROBIS: Risk of bias in systematic reviews
