# Supplementary material for: Air ambulance outcome measures using Institutes of Medicine and Donabedian quality frameworks: protocol for a systematic scoping review
Source: Syst Rev. 2020 Apr 2;9:72. doi: 10.1186/s13643-020-01316-7 (PMC7118977; doi:10.1186/s13643-020-01316-7)
Supplement: Supplementary file 2 — Additional file 2. Medical Subject Headings (MeSH) terms. [file 13643_2020_1316_MOESM2_ESM.docx]

Additional file 2.

*Data sources and search strategy*

Our search strategy will use relevent vocabulary in text combinations, truncation (*) (e.g., air ambulance*, aeromedical*), with search builder restrictions (e.g., AND, OR, NOT); keyword combination, truncation (e.g., patient outcome*, health service outcome*), with search builder restrictions (e.g., AND, OR, NOT); and Medical Subject Headings (MeSH) decision trees structures and search words.
