# Supplementary material for: Air ambulance outcome measures using Institutes of Medicine and Donabedian quality frameworks: protocol for a systematic scoping review
Source: Syst Rev. 2020 Apr 2;9:72. doi: 10.1186/s13643-020-01316-7 (PMC7118977; doi:10.1186/s13643-020-01316-7)
Supplement: Supplementary file 3 — Additional file 3. Review of selected article format. [file 13643_2020_1316_MOESM3_ESM.docx]

**Additional file 3**. Review of selected article format

| Study | N (Total #) | Setting | Population Intervention | Comparison | Outcome |
| --- | --- | --- | --- | --- | --- |
| Results |  |  | Conclusions |  | Quality Level |
| Metrics: | | | Excluded: |  |  |
| Data linkage methods: | | |  |  |  |
| Limitations: | | |  |  |  |
| Data type: | | |  |  |  |
| Data source: | | |  |  |  |
| Mission type: | | |  |  |  |
| Crew type: | | |  |  |  |
| Funding source: | | |  |  |  |
