# Supplementary material for: Air ambulance outcome measures using Institutes of Medicine and Donabedian quality frameworks: protocol for a systematic scoping review
Source: Syst Rev. 2020 Apr 2;9:72. doi: 10.1186/s13643-020-01316-7 (PMC7118977; doi:10.1186/s13643-020-01316-7)
Supplement: Supplementary file 4 — Additional file 4. Risk of bias in systematic review using ROBIS sample. [file 13643_2020_1316_MOESM4_ESM.docx]

**Additional file 4**. Risk of bias in systematic review using ROBIS sample.

| Study | Phase 2 | | | | Phase 3 | Author note |
| --- | --- | --- | --- | --- | --- | --- |
| Author (year) | 1. Study eligibility criteria | 2. Identification and selection of studies | 3. Data Collection and study appraisal | 4. Synthesis and findings | Risk of bias in the review | Rationale |
| Jane Doe (2019) | + | **-** | **-** | ? | + | Narrative description |
